# Inhibitory effects of HNF4α on migration/maltransformation of hepatic progenitors: HNF4α-overexpressing hepatic progenitors for liver repopulation

**DOI:** 10.1186/s13287-017-0629-8

**Published:** 2017-08-14

**Authors:** Ping Wang, Min Cong, Tianhui Liu, Hufeng Xu, Lin Wang, Guangyong Sun, Aiting Yang, Dong Zhang, Jian Huang, Yameng Sun, Wenshan Zhao, Hong Ma, Jidong Jia, Hong You

**Affiliations:** 1 Liver Research Center, Beijing Friendship Hospital, Capital Medical University, Beijing Key Laboratory of Translational Medicine on Liver Cirrhosis & National Clinical Research Center of Digestive Diseases, Beijing, 100050 China; 2 Municipal Laboratory for Liver Protection and Regulation of Regeneration, Capital Medical University, Beijing, 100069 China

**Keywords:** Hepatic nuclear factor 4α, Hepatic progenitors, Proliferation, Liver repopulation

## Abstract

**Background:**

Although they are expandable in vitro, hepatic progenitors are immature cells and share many immunomarkers with hepatocellular carcinoma, raising potential concerns regarding maltransformation after transplantation. This study investigated the effects of hepatic nuclear factor (HNF) 4α on the proliferation, migration, and maltransformation of hepatic progenitors and determined the feasibility of using these manipulated cells for transplantation.

**Methods:**

The effects of HNF4α on rat hepatic progenitors (i.e. hepatic oval cells) were analyzed by HNF4α overexpression and HNF4α shRNA. Nonobese diabetic/severe combined immunodeficiency (NOD/SCID) mice injured by carbon chloride (CCl_4_) were then transplanted with control, HNF4α-overexpressing or HNF4α-suppressing hepatic oval cells. Finally, the engraftment of these cells in the recipient liver was analyzed.

**Results:**

Rat hepatic progenitors (i.e. hepatic oval cells) expressed HNF4α, although less than that in hepatocytes. When HNF4α was overexpressed in these cells, the proliferation and migration of hepatic oval cells were reduced; but when HNF4α was suppressed by shRNA, the proliferation and migration, and even anchorage-independent growth, of these cells were accelerated. RNA microarray and gene functional analysis revealed that suppressing HNF4α not only impaired many biosynthesis and metabolism pathways of hepatocytes but also increased pathways for cancer. When transplanted into CCl_4_-injured NOD/SCID mice, few HNF4α-suppressing hepatic oval cells localized into the liver, while control cells and HNF4α-overexpressing cells engrafted into the liver and differentiated into albumin-positive hepatocytes. Interestingly, the hepatocytes derived from HNF4α-overexpressing cells were less migrative and expressed less c-Myc than the cells derived from control cells.

**Conclusion:**

HNF4α constrains proliferation, migration, and maltransformation of hepatic progenitors, and HNF4α-overexpressing hepatic progenitors serve as an optimal candidate for cell transplantation.

**Electronic supplementary material:**

The online version of this article (doi:10.1186/s13287-017-0629-8) contains supplementary material, which is available to authorized users.

## Background

Orthotropic liver transplantation is the ultimate therapy for patients with end-stage liver diseases but is limited by the shortage of donor organs and the expensive, invasive surgery. Development of cell therapy is critical for treating acute or chronic liver failure and liver-based inherited metabolic disorders [1, 2]. Unfortunately, the availability of high-quality hepatocytes is also limited by organ shortage, and the procedures for isolation, cryopreservation, and cultivation of hepatocytes have detrimental effects on cell viability, metabolic functions, and attachment properties of hepatocytes [3, 4]. Hepatic progenitors with a bipotential signature (i.e. proliferation capacity and differentiation potential) could be long-term expanded in vitro with characteristic stability and genetic integrity [5–7], thus providing a renewable cell source for transplantation. Yet the activation of hepatic progenitors precedes most of the carcinogenic process in chemically induced hepatocellular carcinoma (HCC) animal models, and correlates with the degree of inflammation and stage of fibrosis in human chronic liver diseases [8–11], raising concerns of carcinogenesis and complicating the usage of hepatic progenitors for cell transplantation. Therefore, it is a critical issue to constrain proliferation and prevent maltransformation of hepatic stem/progenitor cells to optimize their clinical usage.

Among the liver-enriched transcription factors we have known, hepatic nuclear factor (HNF) 4α, which acts as a master regulator of liver morphogenesis and hepatocyte differentiation, has anti-proliferation and tumor-suppression functions in the liver [12–15]. Therefore, in the present study we investigated the effect of HNF4α on rat hepatic progenitor cells (also called hepatic oval cells) and determined the feasibility of using these HNF4α manipulated cells for transplantation in carbon chloride (CCl_4_)-injured nonobese diabetic/severe combined immunodeficiency (NOD/SCID) mice.

## Methods

### Detection of HNF4α expression in hepatic progenitors

Hepatic oval cells were isolated by collagenase perfusion and discontinuous gradient centrifugation from rats fed with a choline-deficient diet supplemented with ethionine [7]. The immunophenotype of hepatic oval cells was analyzed by flow cytometry after immunofluorescence staining as described in Additional file 1: Methods. The expression of HNF4α in hepatic oval cells was detected by real-time PCR (RT-PCR) using the isolated rat hepatocytes, cholangiocytes, hepatic stellate cells, endothelial cells, and hepatic oval cells as described in Additional file 1: Methods.

### Manipulation of hepatic oval cells by overexpressing or suppressing HNF4α

HNF4α-overexpression plasmids were constructed by amplifying the *HNF4*α gene from the rat genome DNA by PCR with the forward primer 5′-ataagcttgacatggacatggctgacta-3′ (*Hind*III site underlined) and the reverse primer 5′-atggtaccctagatggcttcctgcttgg-3′ (*Kpn*I site underlined). This 1401-bp rat *HNF4*α gene was inserted into the *Hind*III/*Kpn*I sites of EGFP-N1 vector (BD Biosciences Clontech, Palo Alto, CA, USA) and confirmed by DNA sequencing, forming a recombinant HNF4α plasmid. The HNF4α plasmids were transfected to hepatic oval cells by Lipofectamin 3000 (Invitrogen, Carlsbad, CA, USA) according to the manufacturer’s instructions. Two days post transfection, flow cytometry was used for sorting out EGFP-positive cells according to the method described previously [16], and the sorted cells were cultured in the presence of G418 (Invitrogen) antibiotic selection at 200 μg/ml for 18 days. HNF4α shRNA and negative control, noneffective shRNA were obtained from Santa Cruz (Dallas, TX, USA) and transfected to hepatic oval cells by Lipofectamin 3000 (Invitrogen) according to the manufacturer’s instructions. Two days post transfection, the cells were selected by Puromycin (Santa Cruz) antibiotic selection at 1 μg/ml for 18 days.

### Proliferation and migration of hepatic oval cells after HNF4α overexpression or suppression

Proliferation capacity of hepatic oval cells at 4 days post transfection of the EGFP vector, HNF4α plasmids, noneffective shRNA, or HNF4α shRNA was analyzed by RT-PCR and/or western blot analysis for proliferating cell nuclear antigen (PCNA) and cyclin D1 (CCND1) as described in Additional file 1: Methods. Migration capacity was analyzed by scratch wound healing assay and the anchor-independent growth capacity was detected with soft agar according to the methods described in Additional file 1: Methods.

### Gene expression of HNF4α-overexpressing or HNF4α-suppressing hepatic oval cells

Gene expression profiling of hepatic oval cells (5 × 10^6^) at 4 days post transfection of EGFP vector, HNF4α plasmids, or HNF4α shRNA was analyzed according to the methods described previously [17].

### Transplantation of HNF4α-overexpressing or HNF4α-suppressing hepatic oval cells to CCl_4_-injured NOD/SCID mice

Six-week-old female NOD/SCID mice (body weight (B.W.) around 22 g) were obtained from Beijing HFK Bioscience Co. (Beijing, China) and housed under 12/12-hour light/dark cycles with free access to standard pelleted chow and water. The Animal Care and Use Committees at Beijing Friendship Hospital, Capital Medical University, approved the protocols of these animal experiments. Liver injury was induced by intraperitoneal injection of CCl_4_ (Sigma-Aldrich, St Louis, MO, USA) in olive oil (1:9, v/v; Sigma-Aldrich) at a dose of 0.1 ml/20 g B.W. twice per week for 2 weeks. Two days post final CCl_4_ injection, mice received phosphate-buffered saline (PBS, *n* = 5), 1 × 10^6^ EGFP-vector (*n* = 5), HNF4α-plasmid (*n* = 6) or HNF4α-shRNA (*n* = 3) transfected hepatic oval cells at 4 days post transfection by tail vein injection. The mice were sacrificed at 2 weeks after cell transplantation to collect tissue. Engraftment of cells was verified by double immunofluorescence of ALB and/or GFP.

### Detection of proliferation, migration, and transformation markers in the engrafting cells

Liver tissues with detectable engrafting EGFP-vector or HNF4α-plasmid transfected hepatic oval cells were double-immunostained with ALB and Ki-67 for proliferation analysis, or ALB and MMP2 for migration analysis, or ALB and c-Myc for transformation analysis.

### Statistical analysis

All cell counts were performed on blind-coded samples. Both the total cell numbers (at least 500) based upon DAPI-positive nuclei and the numbers of cells based upon immunoreaction to different markers within the same field were counted from three independent experiments. Data are expressed as the mean ± SD. All comparisons for the study were performed by Student *t* test with SPSS version 16.0. *P* < 0.05 was considered statistically significant.

## Results

### Hepatic oval cells express HNF4α, yet at lower levels than hepatocytes

**Fig. 1:**
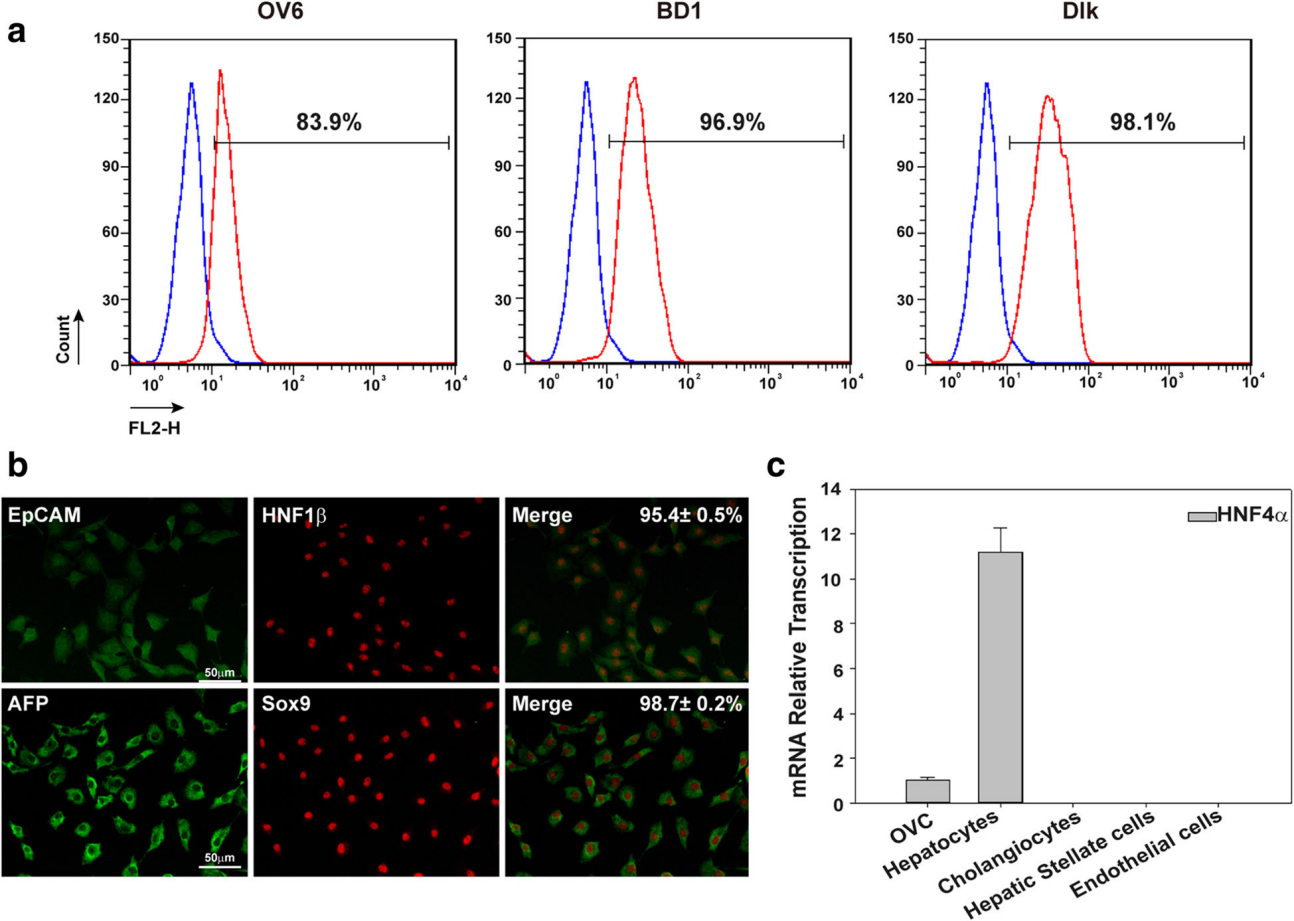
Hepatic progenitors expressed liver-enriched transcription factor HNF4α. **a** Immunofluorescence staining and flow cytometry analysis revealed that isolated rat hepatic oval cells expressed OV6, BD1, and Dlk. **b** Immunophenotype of hepatic oval cells was further revealed by double immunofluorescence staining for EpCAM and HNF1β or AFP and Sox9. **c** RT-PCR analysis revealed that rat hepatic oval cells (OVC) expressed HNF4α, yet to a lesser extent compared to rat hepatocytes. *AFP* α-fetoprotein, *EpCAM* epithelial cell adhesion molecule, *HNF* hepatic nuclear factor, *Sox9* SRY-related HMG box transcription factor 9 (Color figure online)

In order to investigate HNF4α expression in hepatic progenitors and further analyze the effects of HNF4α on hepatic progenitors, rat hepatic progenitors (also called hepatic oval cells) were successfully isolated and their progenitor phenotype was confirmed by 83.9%, 96.9%, and 98.1% positive for markers of hepatic oval cells OV6, BD1, and Dlk (Fig. 1a). These cells are also double positive for the markers of hepatic stem/progenitor cells; that is, epithelial cell adhesion molecule (EpCAM) and HNF-1β or α-fetoprotein (AFP) and SRY-related HMG box transcription factor 9 (Sox9) (Fig. 1b). RT-PCR analysis showed that cholangiocytes, hepatic stellate cells, and endothelial cells did not express HNF4α, but hepatic oval cells and hepatocytes expressed HNF4α, although the expression of HNF4α in hepatic oval cells was at lower levels than that in hepatocytes (Fig. 1c).

### Overexpression of HNF4α reduces the proliferation and migration of hepatic oval cells

**Fig. 2:**
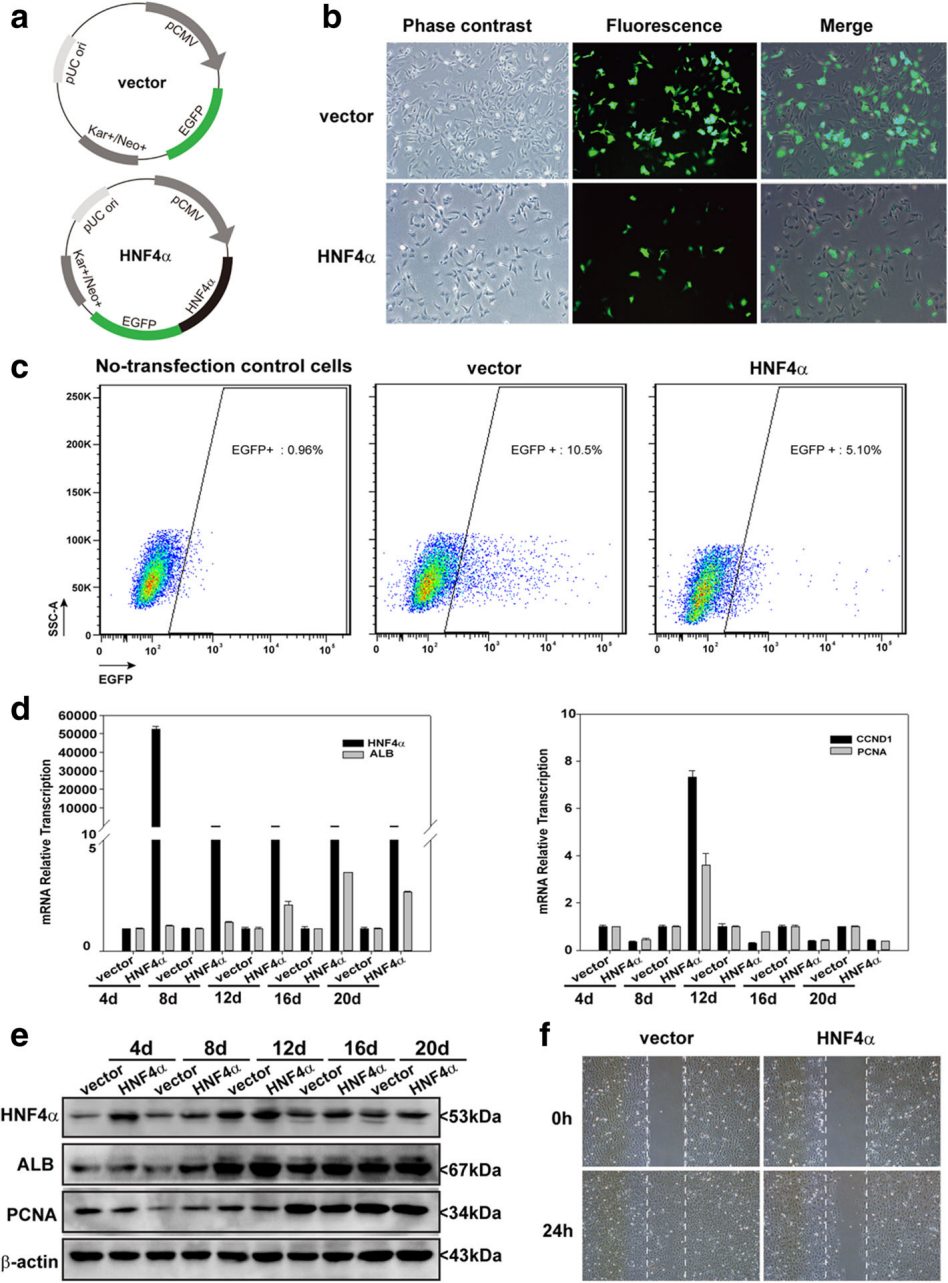
HNF4α overexpression suppressed the proliferation and migration of hepatic oval cells. **a** Recombinant HNF4α-overexpression plasmid and empty EGFP-N1 vector. **b** Forty-eight hours post transfection of HNF4α or EGFP-N1 plasmids, EGFP fluorescence was detected in some hepatic oval cells. **c** HNF4α-overexpressing hepatic oval cells isolated by flow cytometry. **d** RT-PCR data revealed that HNF4α-overexpressing hepatic oval cells showed more transcription of HNF4α and ALB, yet less transcription of CCND1 and PCNA. **e** Western blot analysis confirmed that HNF4α-overexpressing hepatic oval cells expressed more HNF4α and ALB, yet less PCNA. **f** Wound closure was photographed and evaluated at 0 and 24 hours post scratching of HNF4α-overexpressing cells and EGFP-N1 control cells. Overexpression of HNF4α reduced the speed of wound closure when compared to EGFP-N1 control cells. *ALB* albumin, *CCND1* cyclin D1, *HNF* hepatic nuclear factor, *PCNA* proliferating cell nuclear antigen (Color figure online)

HNF4α-overexpression plasmids were constructed (Fig. 2a) and transfected into hepatic oval cells as revealed by green fluorescence at 2 days post transfection (Fig. 2b). The cells with green fluorescence were then sorted out by flow cytometry (Fig. 2c) and cultured in the presence of G418 for 18 days. The overexpression of HNF4α was confirmed by real-time PCR and western blot analysis at 4, 8, 12, 16, and 20 days post transfection (Fig. 2d, e). HNF4α overexpression increased the transcription and expression of ALB, but reduced the expression of PCNA and cyclin D1, when compared to the EGFP-vector transfected control cells at 4, 12, 16, and 20 days post transfection (Fig. 2d, e)—although there is a slight variation for the transcription of PCNA and cyclin D1 under the selection of G418 at 8 days post transfection, which may be interfered with by the dying unsuccessfully transfected cells. Moreover, HNF4α overexpression resulted in a delay in wound closure induced by scratching when compared to EGFP-N1 transfected cells (Fig. 2f).

### Inhibition of HNF4α enhances the proliferation and migration of hepatic oval cells

**Fig. 3:**
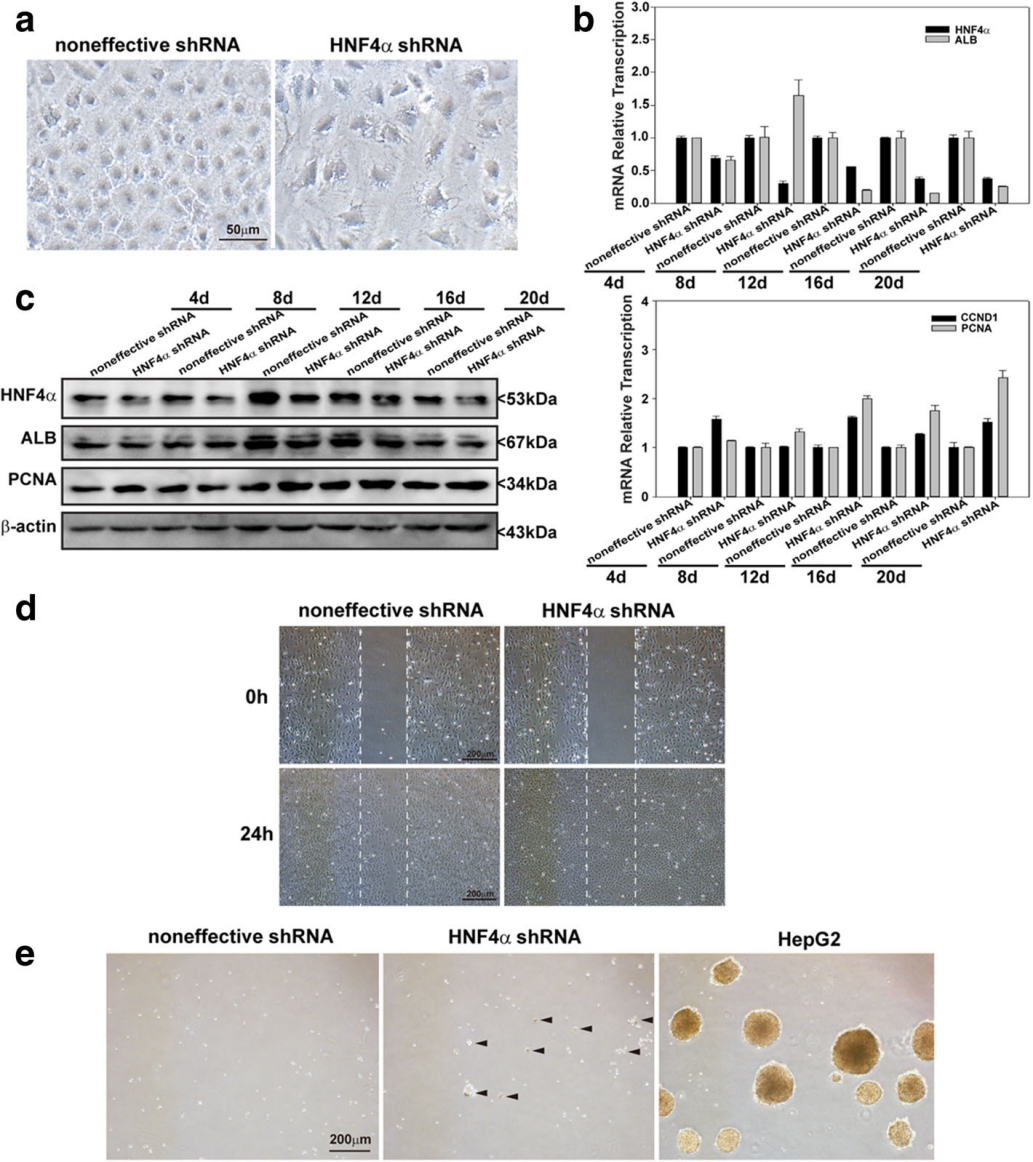
HNF4α suppression accelerated the proliferation and migration of hepatic oval cells. **a** Morphology of hepatic oval cells at 4 days post transfection of noneffective control shRNA and HNF4α shRNA). **b** RT-PCR data showed that HNF4α shRNA transfected cells expressed less HNF4α and ALB, but more CCND1 and PCNA than noneffective control shRNA transfected cells. **c** Western blot analysis confirmed that HNF4α shRNA transfected cells expressed less HNF4α and ALB, but more PCNA than noneffective control shRNA transfected cells. **d** Speed of wound closure was accelerated in HNF4α shRNA transfected cells when compared to noneffective control shRNA transfected cells. **e** Hepatic oval cells transfected with noneffective control shRNA did not proliferate in soft agar after 2-week cultivation, but HNF4α shRNA transfected cells formed some tiny clones in soft agar, although much smaller than HepG2 cells. *ALB* albumin, *CCND1* cyclin D1, *HNF* hepatic nuclear factor, *PCNA* proliferating cell nuclear antigen

Compared with noneffective shRNA transfected hepatic oval cells, HNF4α-shRNA transfected cells showed obvious morphology changes with more cell filaments and reduced nuclear to cytoplasm ratio (Fig. 3a). The expression of HNF4α was reduced at 4, 8, 12, 16, and 20 days post transfection as revealed by RT-PCR and western blot analysis (Fig. 3b, c). Inhibition of HNF4α increased the expression of PCNA and cyclin D1, but reduced the expression of ALB in shHNF4α transfected cells as compared to noneffective shRNA transfected cells at 4, 12, 16, and 20 days post transfection (Fig. 3b, c), and there was also a slight variation for the transcription of ALB, PCNA, and cyclin D1 under the selection of puromycin at 8 days post transfection. Furthermore, suppressing HNF4α increased the wound closure induced by scratching when compared to noneffective shRNA transfected cells (Fig. 3d). In addition, suppression of HNF4α caused hepatic oval cells to proliferate in soft agar although much slower than positive control HepG2 cells, while there was no proliferating signature of the noneffective shRNA transfected cells (Fig. 3e).

### HNF4α contributes to the hepatocyte function pathways and restricts the maltransformation pathway of hepatic progenitors

**Fig. 4:**
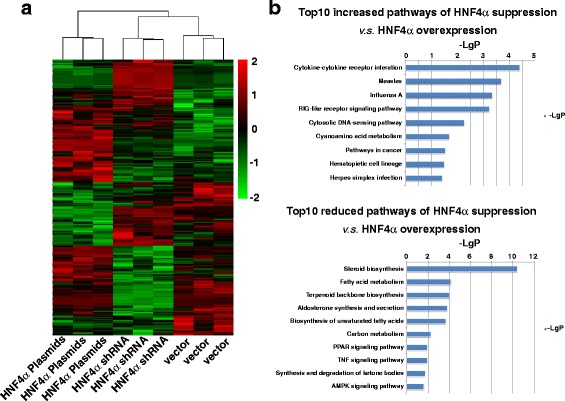
Differential gene expression of hepatic oval cells, HNF4α-overexpressing oval cells, and HNF4α-suppressing oval cells. **a** Hierarchical clustering analysis based on 200 genes significantly differentially expressed between hepatic oval cells, HNF4α-overexpressing oval cells, and HNF4α-suppressing oval cells. Scaled expression values shown for each group; *light green* being the lowest and *light red* the highest expression level. **b** Top 10 upregulated (*upper panel*) and downregulated (*lower panel*) pathways in HNF4α suppression oval cells compared to HNF4α-overexpression oval cells. *HNF* hepatic nuclear factor (Color figure online)

To explore the gene expression variations of HNF4α on hepatic oval cells, whole transcript expression analysis was performed on cells transfected with HNF4α plasmids or HNF4α shRNA by comparing them to EGFP-vector transfected cells. Because we did not find much difference between EGFP-vector transfected cells and noneffective shRNA transfected cells from the previous experiments, only EGFP-vector transfected cells were used as control cells in the transcript-expression analysis and transplantation experiments. After raw data normalization and probe set summary, the expression values of 30,430 transcript clusters were analyzed for differential expression between cells transfected with HN4α plasmids versus HNF4α shRNA versus EGFP-vector plasmids. Intensities of the top 200 differentially expressed genes were subjected to agglomerative hierarchical clustering (AHC) and the results displayed as a heat map (Fig. 4a). The dendrogram showed that the relative gene expression values distinguish the HNF4α-overexpressing cells from the HNF4α-suppressing cells and the control cells. Signal pathways controlled by HNF4α were analyzed by the array data of the HNF4α-overexpressing cells and HNF4α-suppressing cells on the Database for Annotation, Visualization and Integrated Discovery. Among the top 10 enrichment scores, suppressing HNF4α cells greatly reduced the pathways of biosynthesis (steroid, terpenoid backbone, aldosterone, unsaturated fatty acids) and biometabolism (fatty acid, carbon), while increasing pathways in cancer, compared to HNF4α-overexpressing cells (Fig. 4b).

### HNF4α is essential for hepatic oval cells to engraft and reconstitute CCl_4_-injured NOD/SCID mouse liver

**Fig. 5:**
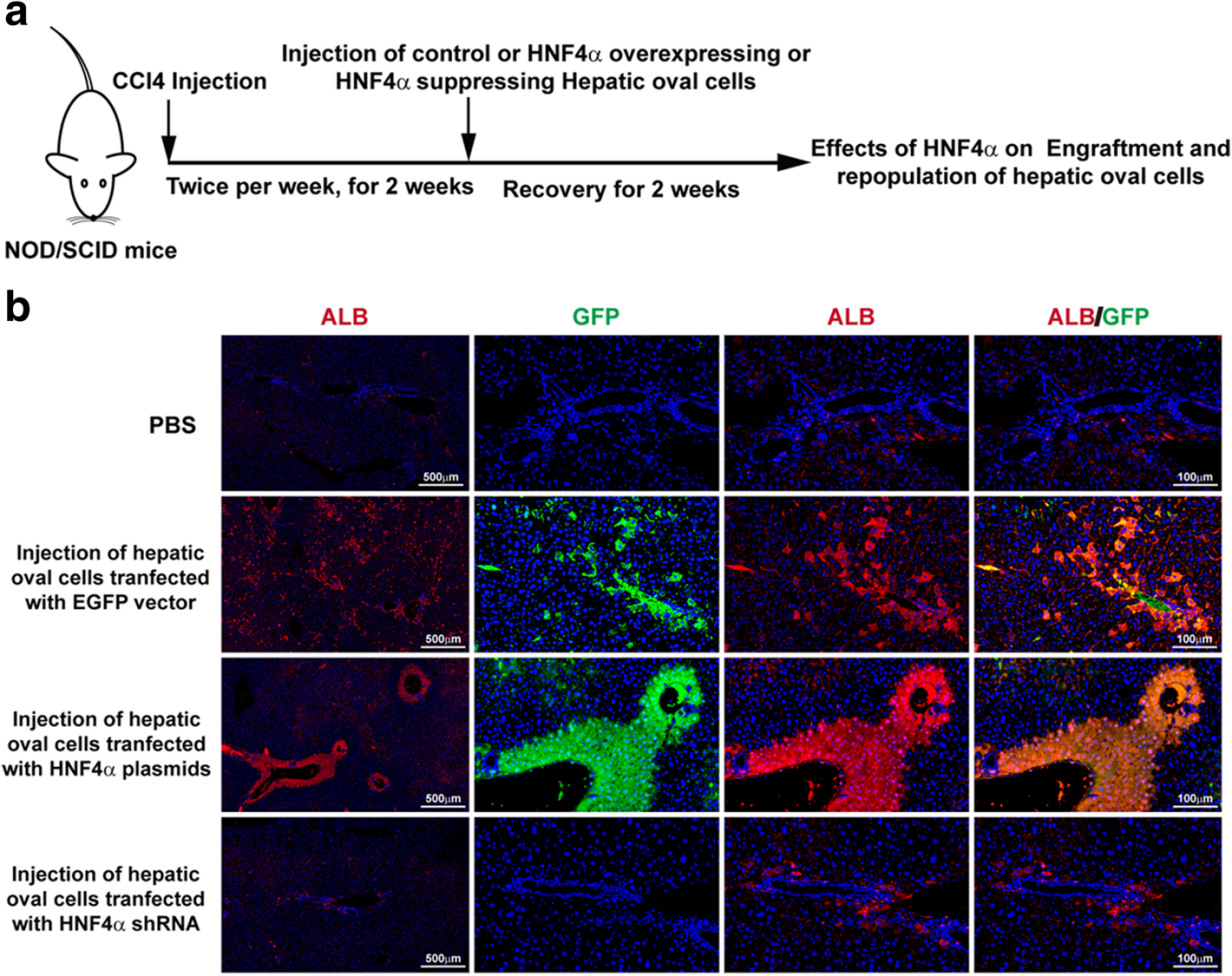
HNF4α was a key transcription factor for hepatic oval cells engrafting and repopulating CCl_4_-injured NOD/SCID mouse liver. **a** Experimental design of transplanting control, HNF4α-overexpressing, or HNF4α-suppressing rat hepatic oval cells into CCl_4_-injured NOD/SCID mice by tail vein injection. **b** Immunofluorescence data revealed that few ALB-positive cells could be detected in HNF4α-suppressing hepatic oval cell-transplanted mice, while ALB and GFP double-positive cells could be detected in the mice transplanted with control and HNF4α-overexpressing hepatic oval cells. Hepatocytes derived from HNF4α-overexpressing oval cells were located around the portal vein, while hepatocytes derived from control oval cells migrated into the recipient liver parenchyma. *ALB* albumin, *CCl*_*4*_ carbon chloride, *HNF* hepatic nuclear factor (Color figure online)

Hepatic oval cells, which were obtained from rat and transfected with EGFP vector, HNF4α plasmids, or HNF4α shRNA, were transplanted into CCl_4_-injured NOD/SCID mice by tail vein injection (scheme of experimental design shown in Fig. 5a). Expression of rat-specific ALB and/or EGFP in the mice liver could localize the cells derived from the transplanted cells. As shown in Fig. 5b, few rat ALB-positive cells could be detected in the mouse liver after transplanting HNF4α-suppressing hepatic oval cells to CCl_4_-injured NOD/SCID mice (*n* = 3), which was similar to CCl_4_-injured mouse liver injected with PBS instead of cells (*n* = 5). However, rat ALB and EGFP double-positive hepatocytes could be detected in the mice transplanted with EGFP-vector transfected control (four of five mice, *n* = 5) or HNF4α-overexpressing (two of six mice, *n* = 6) hepatic oval cells (Fig. 5b), indicating that suppression of HNF4α resulted in defects for hepatic progenitors engrafting into the liver parenchyma (i.e., HNF4α is a pivotal transcription factor for the engraftment of hepatic progenitors).

### HNF4α restricts migration and reduces c-Myc expression in the engrafting hepatic oval cells

**Fig. 6:**
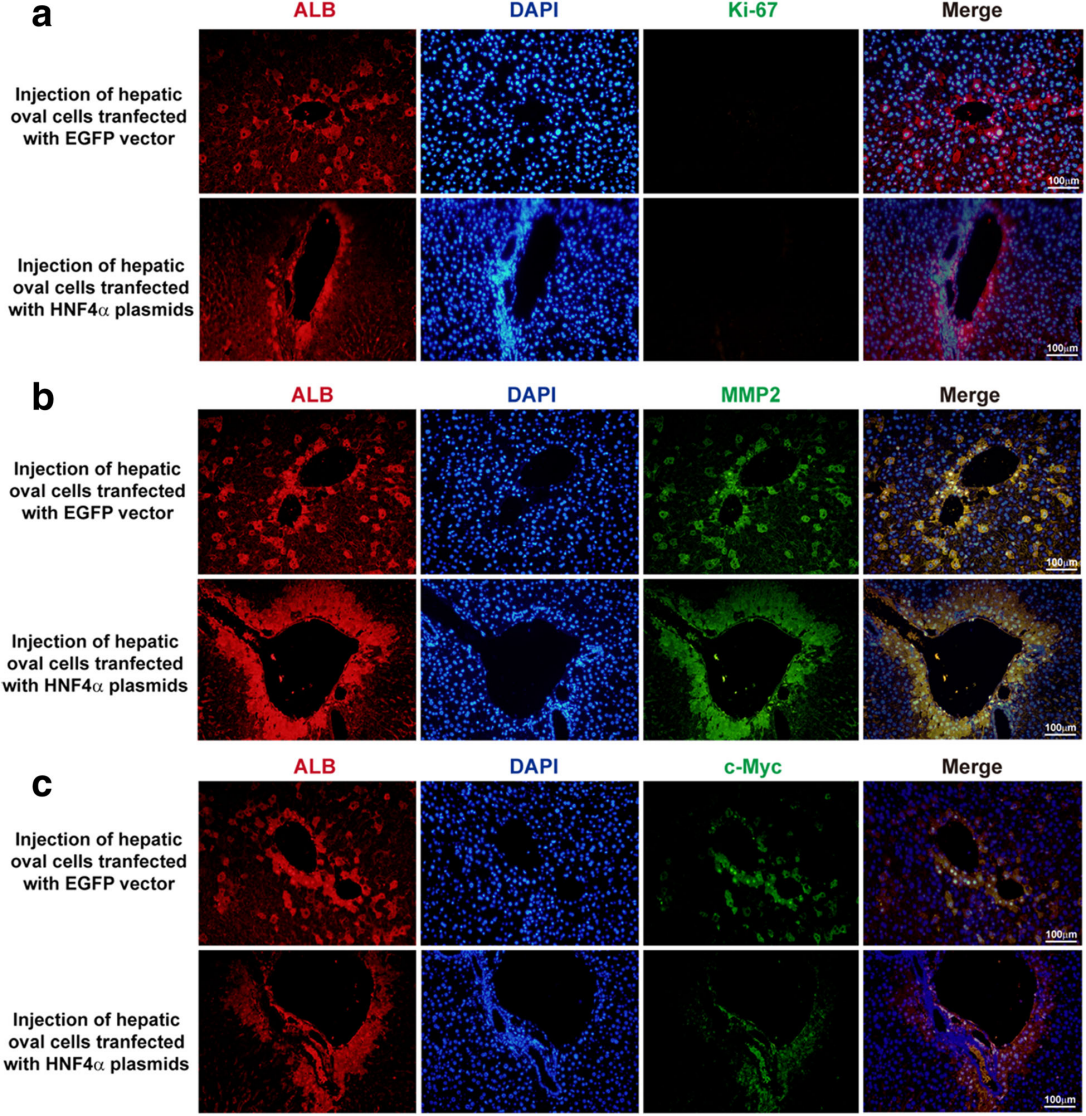
HNF4α reduced expression of c-Myc in the engrafting hepatic oval cells. **a** Double immunofluorescence staining of ALB and Ki-67 showed no Ki-67-positive cells detected in the engrafting cells derived from either HNF4α-overexpressing hepatic oval cells or EGFP-vector transfected controls. **b** Engrafting cells derived from HNF4α-overexpressing hepatic oval cells and EGFP-vector transfected controls expressed MMP2. **c** Engrafting cells around the portal vein, derived from EGFP-vector transfected hepatic oval cells, intensively expressed c-Myc (nuclear staining), while the cells derived from HNF4α-overexpressing cells were weakly positive for c-Myc, although most of them were around the portal vein. When the engrafting cells derived from EGFP-vector transfected hepatic oval cells migrated into the lobule, they no longer express c-Myc. *ALB* albumin, *HNF* hepatic nuclear factor (Color figure online)

**Fig. 7:**
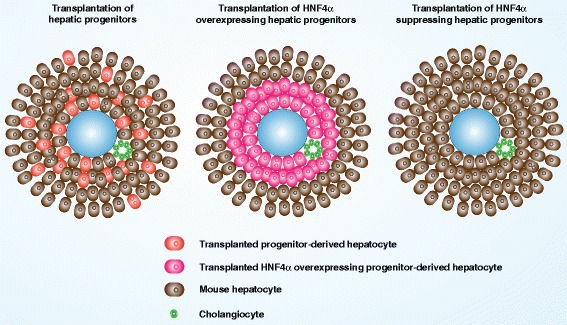
Proposed engraftment and reconstitution models of hepatic progenitors with different HNF4α expression levels. *HNF* hepatic nuclear factor (Color figure online)

For the cells that engrafted into the liver parenchyma, the hepatocytes derived from HNF4α-overexpressing hepatic progenitors settled around the portal vein (identified by the bile duct cells around it), in contrast to the hepatocytes derived from control hepatic oval cells that migrated distally from the portal vein and distributed in the lobule (Fig. 5b). Double immunofluorescence staining of ALB and Ki-67 or ALB and MMP2 revealed that the engrafting cells, derived either from HNF4α-overexpressing hepatic oval cells or from EGFP-vector transfected controls, did not express the proliferation marker Ki-67 (Fig. 6a), but the engrafting cells from both groups expressed MMP2 (Fig. 6b). Furthermore, some of the engrafting cells derived from EGFP-vector transfected control cells were intensively positive for c-Myc, while c-Myc expression was weak in those cells derived from HNF4α-overexpressing cells (Fig. 6c). It is noteworthy that most of the c-Myc-positive cells were around the portal vein, while the cells which migrated into the lobule no longer expressed c-Myc (Fig. 6c). Therefore, these data suggest that the HNF4α expression level determined different reconstitution models of hepatic progenitors (Fig. 7), and although overexpression of HNF4α reduced the engraft efficiency of hepatic oval cells, overexpression of HNF4α restricted the migration and reduced the expression of transformation marker in hepatic oval cells.

## Discussion

In this study, we find that HNF4α constrains proliferation/migration capacity, thus reducing the maltransformation possibility of hepatic progenitors. Furthermore, HNF4α is an essential transcription factor for localizing hepatic progenitors to the injured liver, and different HNF4α expressing levels result in different liver reconstitution models of the transplanted hepatic progenitors, suggesting the feasibility of using HNF4α-manipulated hepatic stem/progenitor cells for transplantation.

Overexpression of HNF4α reduces proliferation/migration capacity of hepatic progenitors, while suppression of HNF4α results in maltransformation signatures. Our results are in agreement with the data that HNF4α suppresses hepatocyte proliferation [13], and are also consistent with the data reporting that inhibition and/or deletion of HNF4α results in promoting hepatocyte proliferation and hepatocellular carcinogenesis, confirming the anti-proliferation and anti-tumorigenesis effects of HNF4α [14, 18, 19]. Thus, manipulation expandable hepatic progenitors through HNF4α overexpression may relieve the concern of maltransformation and provide a sufficient supply of cells for transplantation.

It has been demonstrated that transplantation of hepatic progenitors, which lack sufficient maturity, could reconstitute liver tissue [20–22]. Our study steps further to overexpress HNF4α in hepatic progenitors to restrict proliferation/migration and relieve maltransformation concerns. Although both unmanipulated and HNF4α-overexpressing hepatic progenitors could repopulate the liver parenchyma with ALB-positive hepatocytes, transplantation of HNF4α-overexpressing cells is superior to that of unmanipulated cells in that hepatocytes derived from HNF4α-overexpressing progenitors are less migratory and settle around the portal vein, while the hepatocytes derived from unmanipulated cells migrate into the liver lobule. The reconstitution model of HNF4α-overexpressing cells is similar to the hepatocyte islands or foci derived from mouse or human hepatic stem/progenitor cells, which have been organoid cultivated to induce hepatocyte differentiation, after splenic transplantation into FAH^–/–^ mice or Balb/c nude mice treated with CCl_4_-retrotsine [5, 6]. Considering the migration marker MMP2 is not differentially expressed in the engrafting cells derived from hepatic progenitor cells and HNF4α-overexpressing progenitor cells, there might be some other factors that restrict the migration of HNF4α-overexpressing cells. It has been reported that the larger the cells are, the closer these cells will be to the portal areas after transplantation [23]. The enlarged cell size post HNF4α overexpression may therefore contribute to the different reconstitute models between hepatic progenitors and HNF4α-overexpressing progenitors. Our data reveal that the transformation marker c-Myc is more extensively expressed in the engrafting cells derived from hepatic progenitors around the portal vein than those cells derived from HNF4α-overexpressing cells, although most of the cells derived from HNF4α-overexpressing cells are around the portal vein. Considering c-Myc as an essential transformation marker of aggressive carcinoma cells, the less migratory hepatocytes derived from HNF4α-overexpressing progenitors in vivo reduce the maltransformation possibility of the transplanted cells and provide expandable cells for transplantation.

Of note is that the engraft efficiency of HNF4α-overexpressing hepatic progenitors is not as good as that of unmanipulated hepatic progenitors. Similar results from Zagoura et al. [24] revealed that hepatocyte-like cells fail to engraft into CCl_4_-injured NOD/SCID mice, while hepatic progenitor-like cells incorporate into these mice with therapeutic effects. Yovchev et al. [25] also found that DPPIV^+^ mature hepatocytes reconstitute DPPIV^–^ liver mass to a lesser extent than stem/progenitor cells derived from DPPIV^+^ rat fetal liver. Therefore, it is no surprise to find the lower engraft efficiency for HNF4α-overexpressing hepatic progenitors than unmanipulated progenitors because HNF4α directs them to differentiation toward hepatocytes, although they are not as mature as hepatocytes. Procedures for improving engraft efficiency of HNF4α-overexpressing hepatic progenitors still need further exploration, including the application of vasodilators to help these manipulated cells penetrate through the endothelial barriers.

## Conclusions

HNF4α is an essential transcription factor for restricting the proliferation/migration/maltransformation of hepatic progenitors and controlling their liver repopulation behaviors. HNF4α-overexpressing hepatic progenitors may serve as an alternative cell source for transplantation to treat liver diseases.

## Additional file

Additional file 1: Presents supplemental materials and methods. (DOCX 19 kb)

## Abbreviations

ALB: Albumin
CCl_4_: Carbon chloride
CCND1: Cyclin D1
EpCAM: Epithelial cell adhesion molecule
HNF4α: Hepatic nuclear factor 4α
NOD/SCID: Nonobese diabetic/severe combined immunodeficiency
PCNA: Proliferating cell nuclear antigen
