# Supplementary material for: Inhibitory effects of HNF4α on migration/maltransformation of hepatic progenitors: HNF4α-overexpressing hepatic progenitors for liver repopulation
Source: Stem Cell Res Ther. 2017 Aug 14;8:183. doi: 10.1186/s13287-017-0629-8 (PMC5557474; doi:10.1186/s13287-017-0629-8)
Supplement: Additional file 1: — Presents supplemental materials and methods. (DOCX 19 kb) [file 13287_2017_629_MOESM1_ESM.docx]

**Supplementary Materials:**

**Cell culture**

Hepatic oval cells were isolated by collagenase perfusion and discontinuous gradient centrifugation from rats fed with a choline-deficient diet supplemented with ethionine for 6 weeks as described previously [1]. Hepatic oval cells were cultured at 37˚C with 5% CO_2_ in DMEM/Ham’s F12 (Gibco, Grand Island, NY, USA) supplemented with 10% fetal bovine serum (FBS, Gibco), 1 ng/mL epithelial growth factor (EGF, PeProTech, Rocky Hill, NJ, USA), 0.5 ng/mL stem cell factor (SCF; PeProTech), and 100 U/mL penicillin and streptomycin.

**Immunofluorescence and flow cytometry analysis**

For flow cytometry analysis, cells were detached and fixed with 4% paraformaldehyde in phosphate-buffered saline (PBS) for 20 min. After being permeabilized with 0.1% saponin in PBS and blocked by normal mouse serum, cells were incubated with one of the primary antibodies (mouse anti-rat OV6 antibody, mouse anti-rat bile duct antibody-1 [BD1], or rabbit anti-Dlk antibody [Abcam]) at 37˚C for 60 min. After three washes with 0.1% saponin in PBS, the primary antibodies were detected with corresponding fluorescein isothiocyanate (FITC)-conjugated anti-IgG (BD PharMingen, San Diego, CA, USA). After three washes with 0.1% saponin in PBS, cell fluorescence was analyzed with a FACSCalibur flow cytometer (FACS, Becton Dickinson, Franklin Lakes, NJ, USA) using CellQuest software (BD Bioscience). In the FACS results, the red line represented the positive signals, while the blue line represented isotype as background control.

**Double fluorescence immunocytochemistry**

For immunocytochemistry analysis, cells were fixed with 4% paraformaldehyde in PBS for 20 min. After permeabilization with 0.3% Triton X-100 in PBS and blocking with normal animal serum, the cells were incubated overnight at 4°C with mouse anti-epithelial cell adhesion molecular (EpCAM) antibodies (1:200; Sigma-Aldrich) and rabbit anti-HNF-1β antibodies (1:200; Sigma-Aldrich), or mouse anti-α-fetoprotein antibody (AFP, 1:200; R&D Systems, Minneapolis, MN, USA) and rabbit anti-Sox9 antibodies (1:200, Abcam). After three washes with PBS, the primary antibodies were detected with corresponding Alexa Fluor-conjugated anti-IgG (1:600; Molecular Probes), and the nuclei were counter stained with DAPI.

**RT-PCR**

Freshly isolated rat hepatocytes, cholangiocytes, hepatic stellate cells, and endothelial cells were obtained from OligoBio Biotechnology company (Beijing, China). Total RNA extraction, reverse transcription, polymerase-chain reaction, and data analysis were carried out according to the methods described previously [2]. The primers are listed in the supplemental Table 1, and HPRT gene was used as endogenous reference.

**Western blot**

Standard western blotting procedure was carried out with one of the following primary antibodies: HNF4α antibody (Abcam), ALB antibody (R&D Systems), proliferating cell nuclear antigen (PCNA) antibody (Abcam) and β-actin antibody (Sigma-Aldrich), respectively. Detection of bands was performed using the Molecular Imager Chemi Doc TM XRS+ with Image Lab Software version 3.0 (Bio-Rad, Hercules, CA, USA).

**Scratch wound healing assay**

Wound healing assay was conducted by plating 4X10^5^ cells at 4-day post-transfection of EGFP-N1, HNF4α, shCtrl, or shHNF4α using 35mm culture dishes. When cell confluency was reached after culturing overnight, the cells were wounded with a plastic pipette tip. Photographs were acquired by cellSens Standard software (version 1.6) on Olympus IX71 microscope system (Olympus, Japan) at scratching (0 hour) and after 24 hour.

**Anchorage-independent growth**

The anchorage-independent growth was performed by plating 1X10^4^ hepatic oval cells at 4-day post-transfection of shCtrl, or shHNF4α, or 1X10^4^ HepG2 cells as positive control according to the procedure described previously [2].

**Immunohistochemistry**

Standard immunohistochemistry procedures were carried out according to the methods described previously [3]. Briefly, liver tissues were formalin-fixed and paraffin-embedded. Tissue sections were deparaffinized, rehydrated, and heated with boiling citrate buffer to retrieve the epitopes. After blocking with normal serum, the sections were incubated with rabbit anti-rat albumin (ALB, Bethyl, Montgomery, TX) at 1:200 and one of the primary antibodies [goat anti-green fluorescence protein (GFP, Abcam) at 1:200; sheep anti-Ki-67 (R&D system) at 1:200; goat anti-MMP2 (R&D system) at 1:200; goat anti-c-Myc (R&D system) at 1:200] overnight at 4°C. After three washes in phosphate-buffered saline (PBS) for 5 minutes with gently agitation, the primary antibodies were detected with corresponding horseradish peroxidase labeled (Dako, Glostrup, Denmark) anti-IgG or Alexa Fluor 594 or Alexa Fluor 488 conjugated anti-IgG (1:400; Molecular Probes, Eugene, OR, USA). The nuclei were counterstained with 4’, 6-diamidino-2-phenylindole (DAPI; Sigma-Aldrich, St. Louis, MO, USA).

**Reference:**

1. Wang P, Liu T, Cong M, Wu X, Bai Y, Yin C, et al. Expression of extracellular matrix genes in cultured hepatic oval cells: an origin of hepatic stellate cells through transforming growth factor beta? Liver Int. 2009;29(4):575-584.
2. Wang P, Cong M, Liu TH, Yang AT, Cong R, Wu P, et al. Primary isolated hepatic oval cells maintain progenitor cell phenotypes after two-year prolonged cultivation. J Hepatol. 2010;53(5):863-871.
3. Wang P, Zhang H, Li W, Zhao Y, An W. Promoter-defined isolation and identification of hepatic progenitor cells from the human fetal liver. Histochem Cell Biol. 2008;130(2):375-385.

**Supplement Table 1. Primers Designed for Rat-Specific Genes**

| Name | Accession no. | Primer sequence (5’-3’) | Product (bp) |
| --- | --- | --- | --- |
| ALB | NM_134326 | AGAACCAGGCCACTATCTC | 110 |
|  |  | CAGATCGGCAGGAATGTTGT |  |
| CCND1 | NM_019296 | AAATCCTCCAGGGGATTGTGTTT | 105 |
|  |  | CTGCCAGTTTGATTGTTCCTTTG |  |
| HNF4α | NM_022180 | CACGCGGAGGTCAAGCTAC | 100 |
|  |  | CCCAGAGATGGGGGAGGTGAT |  |
| HPRT | NM_012583 | CCAGTCAACGGGGGACATAAA | 142 |
|  |  | GGGGCTGTACTGCTTGACCAA |  |
| PCNA | NM_022381 | TTGCACGTATATGCCGGGACC | 183 |
|  |  | GCTGAACTGGCTCATTCATCTCT |  |
